# Unique aspects of fiber degradation by the ruminal ethanologen *Ruminococcus albus* 7 revealed by physiological and transcriptomic analysis

**DOI:** 10.1186/1471-2164-15-1066

**Published:** 2014-12-04

**Authors:** Melissa R Christopherson, John A Dawson, David M Stevenson, Andrew C Cunningham, Shanti Bramhacharya, Paul J Weimer, Christina Kendziorski, Garret Suen

**Affiliations:** Department of Bacteriology, University of Wisconsin-Madison, 5159 Microbial Sciences Building, 1550 Linden Drive, Madison, WI 53706-1521 USA; U.S. Dairy Forage Research Center, U.S. Department of Agriculture-Agricultural Research Services, (USDA-ARS), Madison, WI 53706 USA; Department of Biostatistics and Medical Informatics, University of Wisconsin-Madison, Madison, WI 53706-1521 USA

**Keywords:** *Ruminococcus albus*, Cellulose utilization, Ethanol production

## Abstract

**Background:**

Bacteria in the genus *Ruminococcus* are ubiquitous members of the mammalian gastrointestinal tract. In particular, they are important in ruminants where they digest a wide range of plant cell wall polysaccharides. For example, *Ruminococcus albus* 7 is a primary cellulose degrader that produces acetate usable by its bovine host. Moreover, it is one of the few organisms that ferments cellulose to form ethanol at mesophilic temperatures *in vitro*. The mechanism of cellulose degradation by *R. albus* 7 is not well-defined and is thought to involve pilin-like proteins, unique carbohydrate-binding domains, a glycocalyx, and cellulosomes. Here, we used a combination of comparative genomics, fermentation analyses, and transcriptomics to further clarify the cellulolytic and fermentative potential of *R. albus* 7.

**Results:**

A comparison of the *R. albus* 7 genome sequence against the genome sequences of related bacteria that either encode or do not encode cellulosomes revealed that *R. albus* 7 does not encode for most canonical cellulosomal components. Fermentation analysis of *R. albus* 7 revealed the ability to produce ethanol and acetate on a wide range of fibrous substrates *in vitro*. Global transcriptomic analysis of *R. albus* 7 grown at identical dilution rates on cellulose and cellobiose in a chemostat showed that this bacterium, when growing on cellulose, utilizes a carbohydrate-degrading strategy that involves increased transcription of the rare carbohydrate-binding module (CBM) family 37 domain and the tryptophan biosynthetic operon.

**Conclusions:**

Our data suggest that *R. albus* 7 does not use canonical cellulosomal components to degrade cellulose, but rather up-regulates the expression of CBM37-containing enzymes and tryptophan biosynthesis. This study contributes to a revised model of carbohydrate degradation by this key member of the rumen ecosystem.

**Electronic supplementary material:**

The online version of this article (doi:10.1186/1471-2164-15-1066) contains supplementary material, which is available to authorized users.

## Background

Ruminococci are established members of the gastrointestinal tract (GIT) microbiota of ruminants, hindgut-fermenters [1] and monogastrics such as humans [2, 3]. For example, 6 *Ruminococcus* species are among the 57 bacteria that define the “core gut microbiome” found in 90% of humans [4]. The fibrolytic capabilities of many ruminococci make them key players in the dynamics of gut microbial communities and these bacteria have been linked to activities that influence gastrointestinal health in humans [5–8] as well as fiber degradation in ruminants. In the bovine rumen, ruminococci are major contributors to the conversion of fibrous feeds into the organic acids utilized by the host as nutrients [9, 10]. Importantly, ruminococci account for up to 10% of the 16S rRNA gene copies in the bovine rumen, and play a fundamental role in cellulose degradation [11, 12]. Although *Ruminococcus* isolates from the rumen can hydrolyze crystalline cellulose, their activity on other fibrous substrates has not been well-characterized [13, 14]. Investigating how ruminococci degrade fibers will facilitate our understanding of the role that this group plays in host nutrition. In addition, the fermentative capacity of ruminococci, including ethanol production by *R. albus* 7, could inform industrial efforts to convert cellulosic material into commercially relevant bioproducts.

Among the ruminococci, cellulose digestion is best characterized for *R. flavefaciens* [15, 16]. Fiber adherence in *R. flavefaciens* is mediated in part by multienzyme complexes called cellulosomes. Cellulosomes contain cell-anchored scaffold proteins that coordinate fibrolytic enzymes *via* interlocking dockerin and cohesin domains [16, 17]. The scaffold and fibrolytic enzymes are attached to the substrate by carbohydrate binding modules (CBMs), thus localizing the fibrolytic enzymes and hydrolytic products near the cell surface (for a review see [18]). However, the mechanism of adherence to cellulose for other ruminococci such as *R. albus* is less defined.

Cellulosomes have been suspected in some strains of *R. albus* [19], but studies have failed to identify key cellulosomal components in these bacteria. For example, two cellulases were identified in *R. albus* 8 that lacked dockerin domains [20], but instead contained a unique family 37 CBM found only among *R. albus* strains [21, 22]. Additionally, a Pil-family protein was found to be involved in fiber adherence in *R. albus* 8 [23] leading to the suggestion that a combination of cellulosomes, cell-anchored cellulases, and Type IV pili may be involved in fiber adherence [24]. *R. albus* strains also produce a thick matrix of extracellular polysaccharide, called a glycocalyx, when grown on cellulose [25]. Although details of the glycocalyx composition are known [25, 26], the role of the glycocalyx in fiber degradation has not been established.

Given these observations, we hypothesized that *R. albus* 7 does not utilize cellulosomes to degrade crystalline cellulose. To test this, we compared the recently sequenced genome for this bacterium [27] to the genome sequences of other cellulolytic and non-cellulolytic ruminococci and show the lack of complete canonical cellulosomes. We then performed a global transcriptomic analysis of *R. albus* 7 cultures grown on either cellulose or cellobiose to reveal previously unconsidered aspects of cellulose degradation by this bacterium. Finally, we show that *R. albus* 7 utilizes, and produces ethanol from, a number of complex fibrous substrates *in vitro*. Our results contribute to a revised model for fiber degradation by *R. albus* 7 and underscore the important role that ruminococci may play within the mammalian GIT microbiota.

## Results

### *R. albus*7 and *R. albus*8 are distinct from that of *R. flavefaciens*FD-1

**Figure 1:**
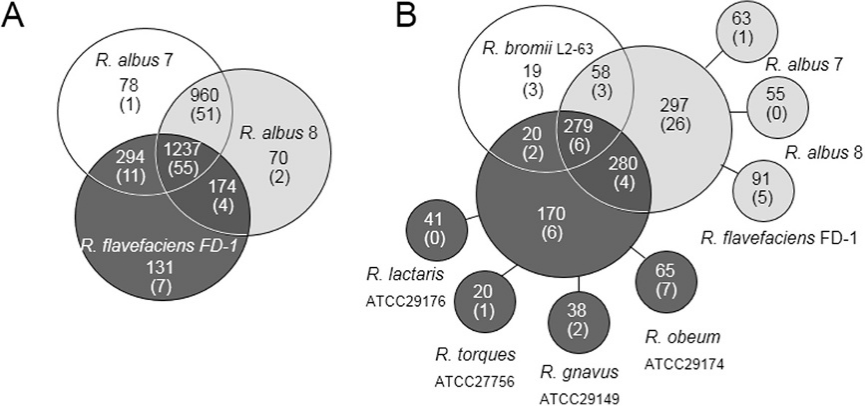
**Functional relationships between sequenced ruminococci. A)** OrthoMCL was used to identify unique and shared proteins among three cellulolytic ruminococci: *R. albus* 7, *R. albus* 8, and *R. flavefaciens* FD-1. Total number of shared orthologous clusters within each group are indicated and the number of orthologous clusters annotated as carbohydrate-active enzymes are shown in parentheses. **B)** Comparison of the three cellulolytic ruminococci (light gray) with non-fibrolytic ruminococci (dark gray), and amylolytic *Ruminococcus bromii* L2-63 (unshaded) emphasized the number of carbohydrate-active enzymes that were specific to these physiologically distinct groups.

To assess the genomic differences between ruminant-derived ruminococci, we compared the genome sequences of three cellulolytic ruminococci: *R. albus* 7, *R. albus* 8, and *R. flavefaciens* FD-1. We employed OrthoMCL, an analysis tool that identifies orthologs by collapsing paralogous proteins and orthologous proteins into orthologous clusters. Using this method, organisms can be differentiated by the presence or absence of clusters. *R. albus* 7 and 8 shared nearly four times as many clusters with each other than with *R. flavefaciens* FD-1 (Figure 1A). Based on a protein family (Pfam) annotation of these clusters, we found that some of the differences between *R. flavefaciens* and the *R. albus* strains included predicted carbohydrate-active enzymes (CAZymes). Overall, we identified 1,234 ortholog clusters common to all 3 bacterial strains, 55 of which contain open reading frames (ORFs) predicted to code for CAZymes. There were few ortholog clusters unique to each organism.

We further refined our CAZyme-containing ortholog clusters to those specific for cellulose utilization by comparing them with five other non-cellulolytic ruminococci (Figure 1B). More than half of the 55 CAZymes common to the cellulolytic ruminococci were shared with non-cellulolytic ruminococci. As expected, many of these predicted CAZymes were involved with general sugar metabolism, such as glycosyl transferases (GT), carbohydrate esterases (CE), or non-cellulolytic families of glycosyl hydrolases (GH) and were therefore not considered further in this study. The remaining CAZyme ortholog clusters are listed in Additional file 1: Table S1 and include numerous cellulases, including three distinct clusters of GH9 cellulases that were common to each of the cellulolytic ruminococci. One of these clusters was similar to the dockerin-containing Cel9A cellulase from *Ruminococcus albus* F-40 [28].

### *R. albus*strains lack most canonical cellulosome components

**Figure 2:**
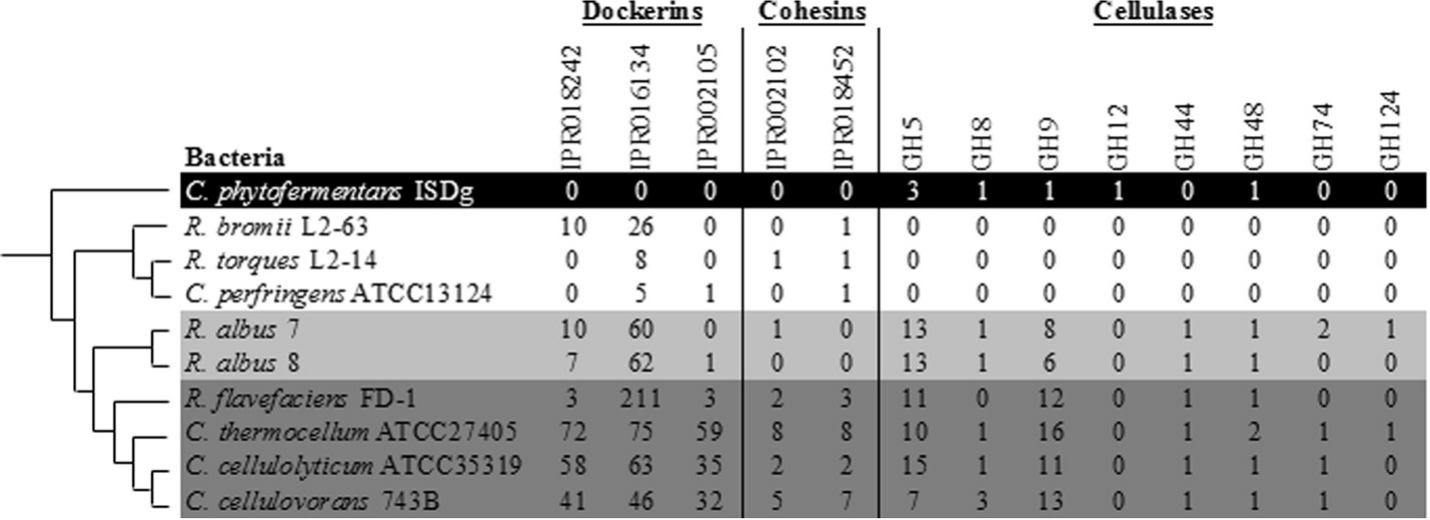
**Comparison of cellulolytic components between cellulolytic and non-cellulolytic bacteria.** Representatives from cellulolytic enzyme families (cellulases) and cellulosome components (dockerins and cohesins) were tabulated in bacteria with unconfirmed cellulosomes (light-gray), confirmed cellulosomes (dark gray), and non-cellulolytic bacteria (unshaded). Sums in each category were used to generate a distance matrix that distinguished groups of these bacteria. Although the cellulolytic *Clostridium phytofermentans* ISDg does not have a confirmed cellulosome, it is highlighted in black to emphasize its relative distinction from the other groups.

We investigated the relative abundance of core cellulosome components and cellulases within several sequenced Clostridiales genomes. Specifically, we chose organisms from the genera *Ruminococcus* and *Clostridium* that have well-characterized canonical cellulosomes (*C. thermocellum* ATCC 27405*, C. cellulovorans* 743B*, C. cellulolyticum* ATCC 35319*, R. flavefaciens* FD-1), cellulolytic organisms not known to contain cellulosomes (*C. phytofermentans* ISDg, *R. albus* 7, *R. albus* 8), and non-cellulolytic organisms (*C. perfringens* ATCC13124*, R. torques* L2-14*, R. bromii* L2-63). We performed a correlation-based clustering analysis and determined the relative similarity between these bacteria (Figure 2). Despite the known diversity among molecular components of cellulosomes, all organisms with characterized cellulosomes that we tested formed a single group distinct from organisms that lacked characterized cellulosomes or from non-cellulolytic organisms. Dockerins, cohesins, and cellulosomal cellulases were generally lacking among the non-cellulolytic organisms with the exception of the amylolytic *R. bromii* L2-63 that had numerous representatives in two of the dockerin families (IPR018242 and IPR016134). The number of representatives in each of the cohesin and dockerin families and the GH9 family were higher among the organisms with canonical cellulosomes (Figure 2).

The *R. albus* strains we examined did not group with any of the cellulosome-containing or non-cellulolytic organisms. Our analysis showed that both *R. albus* strains encode for dockerin-containing proteins, as has been noted previously [27], but lacked representatives of the dockerin family IPR002105 as well as in both cohesin families. Only one putative cohesin (family IPR002102) was found in *R. albus* 7 (Rumal_2328). Moreover, homologs of recognized scaffoldin proteins from *R. flavefaciens* FD-1 (Sca) and *C. thermocellum* ATCC 27405 (Cip) were absent from the *R. albus* strains.

### *R. albus 7*ferments ethanol and acetate from a wide range of substrates

*R. albus* 7 is known to produce ethanol, acetate, H_2_ and CO_2_ from cellulose in pure culture [29]. We verified this and further tested a number of other polysaccharides by measuring ethanol, acetate, and reducing sugar concentrations in batch culture as shown in Table 1. We found that *R. albus* 7 hydrolyzed and utilized a variety of plant polysaccharides, including cellulose and hemicelluloses (homoxylan from tobacco stalk, 4-*O*-methylglucuronoxylan from larchwood). The strain also fermented the storage polysaccharides lichenan (a mixed polymer of β-1,3-, β-1,4-glucose), glucomannan (a mixed polymer of β-1,4-linked glucose and mannose), and the highly complex polysaccharides from soybeans (Table 1). Although *R. albus* 7 hydrolyzed citrus pectin and inulin (a polymer of fructose) to generate reducing sugars, it did not utilize these substrates as shown by the absence of fermentation products. *R. albus* 7 did not hydrolyze or utilize resistant starch (amylose), complex branched polysaccharides (Type II arabinoglacatan), or the storage glucans curdlan (β -1,3-glucan) and laminarin (a mixed β-1,3-, β-1,6 glucan). *R. albus* 7 produced a similar molar ratio (roughly 3:2) of ethanol:acetate regardless of substrate utilized despite slightly higher production of total fermentation products on lichenan and lower production of fermentation products on soy polysaccharides (Table 1). The yield of ethanol produced by *R. albus* 7 per gram of crystalline cellulose was similar to the amount of ethanol produced by other reported ethanologenic cellulolytic organisms grown in batch culture (Additional file 1: Table S2).

**Table 1:** **Hydrolysis and fermentation of polysaccharides by** ***R. albus*** **7**

Polysaccharide	Ethanol ^1^	Acetate ^1^	Reducing sugars ^2^
Cellulose	3.89 ± 0.20	2.33 ± 0.16	0.3 ± 0.5
Xylan (tobacco stalk)	3.82 ± 0.29	2.24 ± 0.19	19.1 ± 3.3
Xylan (larchwood)	3.07 ± 1.01	1.75 ± 0.84	5.4 ± 0.5
Glucomannan	3.77 ± 0.12	2.56 ± 0.13	6.3 ± 0.7
Lichenan	4.46 ± 0.38	2.70 ± 0.26	< 0.2
Soy polysaccharides	1.22 ± 0.19	0.93 ± 0.19	15.2 ± 4.2
Citrus pectin^3^	-	-	44.1 ± 8.9
Inulin^3^	-	-	54.7 ± 1.6

^1^Product formed during growth (mmol/g dry polysaccharide substrate).

^2^Reducing sugars in supernatant (% of dry polysaccharide substrate).

^3^Substrate was hydrolyzed to produce reducing sugars but was not utilized.

*Continuous culture fermentations.* Steady-state cultures grown on cellulose showed a different fermentation pattern than batch cultures, producing more acetate than ethanol (roughly 5:4) with higher levels of each of these fermentation products. Continuous cultures grown on cellulose consumed 1.97 g cellulose/L, equivalent to 10.94 mM glucose equivalents, and produced 11.4 mM ethanol and 14.8 mM acetate. Continuous cultures grown on cellobiose consumed 4.62 g glucose equivalents/L (25.67 mM) and produced 26.9 mM ethanol and 27.7 mM acetate. Cell protein concentrations from cellulose and cellobiose cultures were 0.185 and 0.315 g/L, equivalent to cell yields of 0.188 and 0.136 g/g glucose equivalent, respectively, assuming cells are 50% protein by dry weight.

### The global *R. albus*7 transcriptome revealed differences in gene expression between cellulose- and cellobiose-grown cultures

To identify components important for cellulose degradation, we compared the transcriptome of *R. albus* 7 grown in either a cellulose- or cellobiose-limited chemostat culture. Growth rate has been reported to influence cellulase expression in other cellulolytic organisms [30, 31], and thus a constant 0.033 h^-1^ dilution rate was used for both cellulose and cellobiose in our substrate-limited chemostat. This allows for direct comparison of the effect of substrate type, independent of the confounding effect of growth rate that would result from batch culture cultivation. Ribosome-free RNA was purified from samples taken from steady-state cultures over three days on each substrate and subjected to high-throughput sequencing using Illumina-based RNA-seq, generating at least 9.9 million reads per sample (Additional file 1: Table S3). In most cases, over 90% of the transcripts could be mapped back to the *R. albus* 7 genome sequence, with the remaining unmapped reads representing sequencing errors or unaligned reads due to SNP variation. Over half of the total transcripts were differentially expressed between cellobiose and cellulose substrates with nearly 28% of predicted proteins increased by two-fold or more in response to growth on cellulose (Additional file 1: Figure S1). This broad transcriptional response spanned the entire chromosome as well as the four plasmids, and contained reproducible expression patterns across each replicate chemostat sample with the exception of one cellobiose sample (Additional file 1: Figure S2). Incompletely digested chromosomal DNA or an RNAase may have contaminated this sample and it was not included in any further analysis. Predicted open reading frames (ORFs) with lower transcription on cellulose, as compared to cellobiose, are described in Additional file 1: Supplementary Information 1 and Table S4.

### Increased transcription of cellulases during *R. albus*7 growth on cellulose compared to cellobiose

Thirteen predicted CAZymes, including cellulases and hemicellulases, exhibited greater than 4-fold higher transcription when grown on cellulose, as compared to cellobiose. All of the 27 predicted cellulases encoded by the *R. albus* 7 genome sequence had higher transcription on cellulose than cellobiose, although only 4 exhibited greater than 4-fold expression (Table 2). These included two GH9 cellulases (Rumal_1569 and Rumal _2448) and two GH5 cellulases (Rumal_0896 and Rumal_2599). Fourteen of the twenty-seven predicted cellulases were not differentially expressed (had a fold-change less than two, data not shown) and none of the predicted cellulases had lower transcription when *R. albus* 7 was grown on cellulose. Using the PRED-TAT software [32], we found that all of the predicted cellulases and hemicellulases contained signal peptides for Sec-dependent secretion (Table 2).

**Table 2:** **Genes with greater than 4-fold higher transcription during growth of** ***R. albus 7*** **on cellulose compared to cellobiose as the sole carbohydrate source assessed by RNA-Seq**

Gene (Rumal_)	Annotation ^1^	Fold Change ^2^	Signal sequence ^3^
1715	Indole-3-glycerol-phosphate synthase	15.50	No
1714	chorismate mutase	15.49	No
1716	anthranilate phosphoribosyltransferase	14.90	No
1712	tryptophan synthase subunit alpha	14.90	No
1713	tryptophan synthase subunit beta	14.10	No
1717	anthranilate synthase component I	13.62	No
3687	transcriptional regulator, ArsR family	11.53	No
3686	protein of unknown function DUF1648	11.32	Sec
0360	helix-turn-helix domain-containing protein	10.04	No
3688	protein of unknown function UCP033101	9.98	TM
2652	putative multiple sugar transport system substrate-binding protein	9.80	Sec
2572	ECF subfamily RNA polymerase sigma-24 subunit	8.82	No
2573	hypothetical protein	8.18	TM
**0897**	**fibronectin type III domain-containing protein (CBM37)**	**7.93**	**Sec**
3780	Resolvase domain	7.91	No
**2448**	**Cellulase (GH9 CBM3 CBM37 CBM37)**	**7.70**	**Sec**
3766	hypothetical protein	7.42	Sec
0953	hypothetical protein	7.00	Sec
3796	ABC transporter family protein	5.91	No
**1569**	**Cellulase (GH9 CBM37)**	**5.56**	**Sec**
3775	ABC transporter family protein	5.53	No
**3757**	**Endo-1,4-beta-xylanase (GH11 CBM22 GH10 CBM37)**	**5.51**	**Sec**
3776	IstB domain protein ATP-binding protein	5.21	No
**0896**	**Cellulase (GH5 CBM37)**	**5.15**	**Sec**
**1952**	**fibronectin type III domain-containing protein (PL1 CBM37)**	**5.00**	**Sec**
3963	hypothetical protein	4.84	TM
1427	hypothetical protein	4.74	TM
**0487**	**Ricin B lectin (CE12 CBM13 CBM35 CE12)**	**4.74**	**Sec**
3179	Capsule synthesis protein CapA	4.68	No
**2739**	**endo-1,4-beta-xylanase (GH11 CBM22 CE1 CBM37)**	**4.63**	**Sec**
**1262**	**fibronectin type III domain-containing protein (PL11 CBM37)**	**4.61**	**Sec**
**0908**	**endo-1,4-beta-xylanase (GH11 CBM22 CE4 CBM37)**	**4.59**	**Sec**
3944	chaperone protein DnaK	4.48	No
1426	hypothetical protein	4.37	Sec
1821	family 1 extracellular solute-binding protein	4.36	Sec
3777	Integrase catalytic region	4.33	No
1428	hypothetical protein	4.30	TM
1602	transaldolase	4.26	No
**2599**	**glycoside hydrolase family 5 (GH5 CBM37)**	**4.23**	**Sec**
3940	hypothetical protein	4.22	No
3798	IS66 Orf2 family protein	4.22	No
3186	family 1 extracellular solute-binding protein	4.18	Sec
**1951**	**fibronectin type III domain-containing protein (CBM37)**	**4.17**	**Sec**
3184	binding-protein-dependent transport systems inner membrane component	4.16	TM
3185	binding-protein-dependent transport systems inner membrane component	4.12	No
3774	hypothetical protein	4.10	No
**1044**	**spore coat protein CotH (CBM37)**	**4.09**	**Tat**
3263	hypothetical protein	4.02	No

^1^Each gene reported here was significantly DE, with a PP of DE greater than 0.95

^2^Predicted signal sequence predicted by PRED-TAT. Sec = Sec-dependent; TM = transmembrane.

^3^Genes containing predicted CBM37 domains are presented in bold.

Of the CAZymes found to be up-regulated on cellulose, relative to cellobiose, all but one contained one or more CBM37 domains. We identified 64 proteins containing one or more CBM37 domains in the *R. albus* 7 genome sequence, and 39 of them had more than 2-fold higher transcription when grown on cellulose, relative to cellobiose. The CAZyme with the highest differential expression (Rumal_0897) contained a single CBM37 as its only predicted CAZy domain. *R. albus* 7 has 35 proteins predicted to contain binding modules with no adjacent catalytic domains, including 26 containing a single CBM37, 8 that each contain 2 CBM37 domains, 1 that contains a CBM37 and a CBM2 domain, 1 that contains 2 CBM37 domains, and 2 containing a CBM13 and a CBM37 domains. In addition to these ORFs, we also investigated whether or not putative pilin-like genes and genes involved in glycocalyx biosynthesis had higher transcription on cellulose, relative to cellobiose, and found that none of these genes were differentially expressed (Additional file 1: Figures S3 and S4).

In addition to CAZymes, ORFs found to be expressed more than 4-fold or higher on cellulose, relative to cellobiose, include those predicted to be involved in transcription, and primary metabolism. There were also several clusters of ORFs that were differentially expressed including four putative ABC transporter operons that each had 4-fold or higher transcription in response to growth on cellulose (Rumal_3686 – Rumal_3688, Rumal_3184 – Rumal_3187, Rumal_3774 – Rumal_3777, and Rumal_1427 – Rumal_1429). Most notably, the entire tryptophan biosynthetic operon had over 13-fold higher transcription in response to cellulose, and is discussed in the following section.

### Tryptophan enrichment among CAZymes is widespread among cellulolytic organisms

The tryptophan biosynthetic operon was the most highly expressed set of ORFs in the *R. albus* 7 chemostat culture grown on cellulose, as compared to cellobiose (Table 2). This finding was further verified using reverse transcription quantitative PCR (RT-qPCR) of the tryptophan biosynthetic gene, Rumal_1716, normalized to 16S rRNA, which was found to be expressed 7.2 fold higher on cellulose, relative to cellobiose. Despite the highly regulated nature of this pathway, induction of the tryptophan operon in response to growth on cellulose has been previously observed in *C. phytofermentans* where it was thought to involve an increased need for tryptophan in the CAZymes important for growth on cellulose [33]. We tested this hypothesis for *R. albus* 7 and found that CAZymes in this bacterium also showed a significantly higher percentage of tryptophans compared to all other genes in the genome (Table 3). An analysis of CAZymes from a range of other cellulolytic, amylolytic and hemicellulolytic species also showed a significant enrichment for tryptophan as well as tyrosine (*P* < 0.01), but not for non-aromatic amino acids like methionine (Table 3). This indicates that a specific enrichment for aromatic amino acids among CAZymes is widespread among cellulolytic bacteria, and not specific to *R. albus* 7 or *C. phytofermentans*.

**Table 3:** **Aromatic amino acid enrichment among CAZymes**

	Percentage Tryptophan ^1^			Percentage Tyrosine ^2^			Percentage Methionine ^2^		
Organism	In all proteins	In CAZymes	P-Value	In all proteins	In CAZymes	P-Value	In all proteins	In CAZymes	P-Value
*Acidothermus cellulolyticus* 11B	1.4	9.7	< 0.01	2.1	9.2	< 0.01	1.6	5.0	0.99
***Actinoplanes missouriensis*** **431**	**1.6**	**10.0**	**< 0.01**	**2.0**	**9.9**	**< 0.01**	**1.8**	**5.5**	**1.00**
***Butyrivibrio proteoclasticus*** **B316**	**0.9**	**16.9**	**< 0.01**	**4.6**	**11.9**	**< 0.01**	**3.0**	**8.6**	**1.00**
*Caldicellulosiruptor bescii* DSM6725	0.9	15.5	< 0.01	4.3	7.9	< 0.01	2.2	5.8	1.00
***Caldicellulosiruptor kristjanssonii*** **177R1B**	**0.8**	**12.8**	**< 0.01**	**4.2**	**7.6**	**< 0.01**	**2.2**	**5.2**	**1.00**
*Caldicellulosiruptor saccharolyticus* DSM8903	0.9	15.4	< 0.01	4.3	8.4	< 0.01	2.2	5.9	1.00
*Cellulomonas fimi* ATCC484	1.6	13.0	< 0.01	1.8	12.3	< 0.01	1.4	6.8	1.00
*Cellulomonas flavigena* DSM20109	1.6	11.0	< 0.01	1.8	11.2	< 0.01	1.4	6.6	1.00
*Cellulomonas gilvus* ATCC13127	1.6	12.0	< 0.01	1.8	11.4	< 0.01	1.5	6.0	1.00
*Cellvibrio japonicus* Ueda107	1.5	15.8	< 0.01	3.2	13.0	< 0.01	2.2	8.1	1.00
*Clostridium acetobutylicum* ATCC824	0.7	15.6	< 0.01	4.3	9.1	< 0.01	2.6	6.3	1.00
*Clostridium cellulolyticum* H10	0.9	163	< 0.01	4.1	9.4	< 0.01	2.6	6.6	1.00
*Clostridium cellulovorans* 743B	0.8	16.5	< 0.01	4.3	9.7	< 0.01	2.4	7.2	1.00
*Clostridium phytofermentans* ISDg	0.9	16.3	< 0.01	4.4	9.7	< 0.01	2.8	6.5	1.00
*Clostridium thermocellum* ATCC 27405	0.9	17.1	< 0.01	4.2	10.3	< 0.01	2.5	6.7	1.00
*Cytophaga hutchinsonii* ATCC33406	1.1	10.6	< 0.01	4.4	7.3	< 0.01	2.2	5.6	1.00
*Fibrobacter succinogenes* S85	1.3	15.3	< 0.01	3.7	11.7	< 0.01	2.6	9.1	1.00
***Flavobacterium johnsoniae*** **UW101**	**1.1**	**12.8**	**< 0.01**	**4.2**	**8.1**	**< 0.01**	**2.1**	**6.8**	**0.97**
*Micromonospora aurantiaca* ATCC27029	1.6	8.6	< 0.01	2.0	8.8	< 0.01	1.6	5.1	1.00
***Prevotella ruminicola*** **23**	**1.4**	**17.9**	**< 0.01**	**4.3**	**12.5**	**< 0.01**	**3.0**	**9.7**	**1.00**
*Ruminococcus albus* 7	0.9	13.8	< 0.01	4.3	8.0	< 0.01	2.9	5.6	1.00
*Ruminococcus albus* 8	0.9	12.5	< 0.01	4.2	7.4	< 0.01	2.9	5.1	1.00
***Ruminococcus bromii*** **L2-63**	**0.7**	**9.0**	**< 0.01**	**4.1**	**5.7**	**< 0.01**	**2.7**	**4.0**	**0.69**
*Ruminococcus flavefaciens* FD1	0.9	15.9	< 0.01	4.4	9.3	< 0.01	2.8	7.0	1.00
***Ruminococcus torques*** **L2-14**	**0.98**	**7.8**	**< 0.01**	**4.0**	**5.4**	**< 0.01**	**3.2**	**3.9**	**1.00**
*Streptomyces flavogriseus* ATCC33331	1.5	8.2	< 0.01	2.1	8.2	< 0.01	1.8	4.4	1.00
*Streptomyces sp.* ACTE	1.5	8.8	< 0.01	2.0	8.5	< 0.01	1.7	4.5	1.00
*Teredinibacter turnerae* T7901	1.4	12.2	< 0.01	3.1)	8.7	< 0.01	2.2	5.7	1.00
*Thermobifida fusca* YX	1.5	7.6	< 0.01	2.2	6.7	< 0.01	1.7	4.1	1.00
*Thermomonospora curvata* DSM43183	1.5	4.1	< 0.01	2.1	4.0	< 0.01	1.9	2.9	0.73
*Trichoderma reesei* QM6a	1.5	6.1	< 0.01	2.7	5.6	< 0.01	2.2	3.8	1.00
*Xanthomonas campestris* ATCC33913	1.6	8.6	< 0.01	2.4	8.0	< 0.01	2.1	5.2	1.00

^1^Non-cellulolytic strains are bolded.

^2^Percentage of selected amino acid in all proteins with percentage of selected amino acid in predicted CAZymes in parenthases. *P*-value based on Fisher’s exact test for the enrichment of the selected amino acid among CAZymes.

Since CAZymes account for only 6.5% of the proteins predicted to be encoded by the genome sequence of *R. albus* 7, a small enrichment (~2-fold) of tryptophan in CAZymes is unlikely to require a 13-fold increase in transcription of the tryptophan biosynthetic operon. This is supported by our finding that tRNA transcription and transcription of other aromatic amino acid biosynthetic pathways, including tyrosine, were unaffected by growth on cellulose (data not shown). An alternative hypothesis is that exogenous tryptophan accumulates in *R. albus* 7 when grown on cellulose. We tested this and found that extracellular tryptophan did not accumulate in *R. albus* 7 batch cultures grown on cellulose (data not shown).

## Discussion

Ruminococci are ubiquitous members of mammalian GIT microbial communities and play an important role in plant fiber degradation in the bovine rumen. As key members of the rumen ecosystem, *Ruminococcus* spp. are known for their ability to degrade a wide range of plant polysaccharides, including cellulose. Here we use both genomic and transcriptomic evidence to show that *R. albus* 7 is distinct from other cellulosome-utilizing ruminococci. Adherence to fiber is required for cellulose hydrolysis by *R. albus* strains and several mechanisms for fiber-digestion have been proposed by others. Importantly, four components have been implicated in *R. albus* fiber adherence including: pilin-like appendages [23], a glycocalyx [25, 26], cellulosomes [19], and the unique carbohydrate binding module CBM37 [21, 22]. These findings led to the model that a combination of these four components may be involved in fiber attachment in *R. albus* [24].

We examined both genomic and transcriptomic evidence for each of the four components implicated in *R. albus* 7 fiber adherence. First, we were able to identify pilin-like genes encoded by a *pil*/*sec* locus in the *R. albus* 7 genome sequence with high similarity and identical organization to the loci identified in *R. albus* 20 and *R. albus* 8 [34]. However, our transcriptional analysis did not detect changes in this locus in response to growth on cellulose (Additional file 1: Figure S3). A molecule required for optimal growth on cellulose, 3-phenylpropanoic acid (PPA), was shown by others to increase transcription of the *pilA1* (*cbpC*) gene in *R. albus* 8 [23]. We did not observe an increase in expression for this ORF in *R. albus* 7 but PPA was present under all growth conditions tested in our experiments.

Second, *R. albus* 7 forms a glycocalyx when grown on cellulose comprised of proteins, uronic acids and sugars including glucose, xylose, some mannose and other sugars [25]. We identified genes in *R. albus* 7 with putative roles in the anabolic sugar pathways for xylose, mannose, and fructose. However, key enzymes in the synthesis of UDP-xylose, such as a UDP-galactose decarboxylase or a glucose-1-phosphate uridyltransferase were not present in the *R. albus* 7 genome (Additional file 1: Figure S4). This is surprising because as much as 20% of the glycocalyx in *R. albus* 7 is composed of xylose [25] and it may indicate that synthesis of UDP-xylose is achieved by a different biosynthetic route, or that UDP is not involved in xylose synthesis. Although glycocalyx biosynthetic genes have not been experimentally identified in *R. albus* 7, none of the anabolic sugar pathway genes with a predicted role in glycocalyx formation had significantly higher transcription during growth on cellulose and a few of these genes had lower transcription during growth on cellulose (Additional file 1: Figure S4). These findings suggest that, at the transcriptional level, glycocalyx synthesis is not induced during growth on cellulose.

Third, microscopic evidence for “cellulosome-like protuberances” in some strains of *R. albus* [19, 26, 35] along with molecular evidence for cellulosomal components in *R. albus* F-40 and *R. albus* SY3 [36, 37] led to the proposal that at least some *R. albus* strains produce cellulosomes. However, several endoglucanases and xylanases in *R. albus* F-40 and *R. albus* SY3 lack dockerin domains and fibrolytic activity in *R. albus* SY3 did not associate with high molecular-weight protein complexes. These results suggested that non-cellulosomal glycanases may also be present in these *R. albus* strains [37]. In our analysis, *R. albus* 7 contained few homologs of known cellulosome components, consistent with previous reports [38]. Scaffoldins, like those found in *R. flavefaciens* FD-1 or *C. thermocellum* ATCC 27405, were entirely absent and only one putative cohesin was identified. This cohesin (Rumal_2328) had an average of 2.72-fold higher transcription when grown on cellulose compared to cellobiose. Moreover, *R. albus* 7 contained fewer dockerins than bacteria with confirmed cellulosomes and most of these belong to the Interpro families IPR018242 or IPR016134, which were also abundant in other non-cellulolytic ruminococci like *R. bromii* L2-63 (Figure 2). This finding could imply a physiological role for these dockerins that is distinct from their role in cellulosomes. In support of this hypothesis, a global analysis of dockerins and cohesins identified their widespread presence among non-cellulolytic organisms in all three domains of life [39]. These non-cellulosomal dockerins and cohesins are also suspected in cellulolytic organisms that form multi-enzyme cellulolytic complexes. For instance, dockerin domains were identified in the *R. flavefaciens* FD-1 genome in ORFs that lacked CAZymes but contained leucine-rich repeat, transglutaminase, and serine protease inhibitor modules instead [15].

Fourth, in the absence of cellulosomes, *R. albus* 7 and *R. albus* 8 may instead rely on alternate carbohydrate-binding modules, such as CBM37s, to facilitate coordination of secreted carbohydrases. This conclusion is supported by our finding that all of the CAZymes with 4-fold or higher transcription on cellulose contained CBM37 domains, with the exception of Rumal_0487. We identified 34 proteins containing one or more CBM37 domains that lacked adjacent catalytic domains. We analyzed these proteins and found that nearly half of them also contained leucine-rich repeat domains. Leucine-rich repeats have been implicated in protein-protein interaction [40], and it is possible that CBM37 functions to coordinate an extracellular complex of carbohydrases. Our results also indicated that, in general, genes that had higher transcription during growth on cellulose were distinct from genes encoding pilin structures and glycocalyx components that have been shown by others [19] to be affected by PPA. Future work will be needed to define these regulons.

The cellulolytic capacity of *R. albus* 7 has historically been correlated to the production of a number of fermentation products like acetate and ethanol [29]. Our analysis expands on these findings to further reveal that *R. albus* 7 can utilize more polysaccharides than other ruminal cellulolytic bacteria like *F. succinogenes* S85 [41], suggesting that it may be more of a carbohydrate generalist in the rumen. Moreover, we found that *R. albus* 7 hydrolyzed several components of the plant cell wall and produced yields of ethanol and acetate comparable to other ethanologenic cellulolytic organisms. Although we observed different ratios of ethanol:acetate in batch *versus* continuous culture, the ethanol:acetate ratio has been shown to vary with dilution rate in continuous cultures of *R. albus* 7 [29]. We found more reducing sugars were produced from homoxylan purified from tobacco stalk than from the more complex larchwood xylan. The difference in reducing sugars likely indicates less fermentation of the larchwood xylan because lower amounts of fermentation products were produced from this substrate. In contrast, we found higher cell yields when *R. albus* 7 was grown on cellulose, relative to cellobiose, and we believe that this observation likely reflects the higher net ATP yield from phosphorolytic cleavage of cellodextrins compared to that on cellobiose, as has been previously proposed [42].

In the rumen, *R. albus* 7 encounters plant biomass that includes complexes of cellulose, hemicelluloses, and other polysaccharides. When grown on crystalline cellulose as the sole carbon source, we found that *R. albus* 7 had a broad transcriptional response that included increased transcription of hemicellulases and other carbohydrases for substrates that were not present in the growth medium. Importantly, the transcriptional differences that we observed were not confounded by growth rate, a factor that is known to affect differential genes expression in other cellulolytic bacteria [30, 31], as the growth of *R. albus* 7 was controlled using the same dilution rate with each substrate. This may indicate that cellulose initiates a generalized transcriptional response to plant fibers in *R. albus* 7, or that cellobiose represses such a response, although this hypothesis remains to be tested.

We were surprised to find that the tryptophan biosynthetic operon was highly transcribed when *R. albus* 7 was grown on cellulose, relative to cellobiose in chemostat cultures. Although increased transcription of tryptophan biosynthesis is not a typical observation for other cellulolytic organisms [30, 31], a similar response has been noted for *C. phytofermentans* ISDg [33]. *C. phytofermentans* ISDg is also an ethanologenic, cellulolytic organism, but is distinguished from *R. albus* 7 by the relative abundance of cohesins, dockerins and cellulases (Figure 2). It is reasonable to propose that increased transcription of the tryptophan operon is due to increased demand for tryptophan in CAZymes, however, we found that tryptophan enrichment (and tyrosine enrichment) in carbohydrate-active enzymes is widespread, even among non-fibrolytic organisms. We did not detect an increase of the tyrosine biosynthetic pathway or any other (non-tryptophan) amino acid biosynthetic pathway in response to growth on cellulose. Based on these findings it is unlikely that the transcriptional increase of the tryptophan biosynthetic pathway could be explained by increased production of CAZymes. Taken together, these results could suggest a role for tryptophan in a peripheral metabolic activity concomitant with, but distinct from, growth on cellulose. For instance, tryptophan may confer ethanol stress tolerance as has been reported for yeast [43]. Alternatively, tryptophan or their derivatives could be used as signaling molecules, as tryptophan-derived tryptophols are reported to serve as quorum-sensing molecules under nitrogen-poor conditions in yeast [44]. A similar mechanism might be employed by *R. albus* 7 to coordinate fiber adherence, but future work will be needed to determine the exact role of tryptophan in cellulose degradation.

## Conclusions

This study provides the first comprehensive transcriptomic analysis for any ruminococcal species, in addition to characterizing the fermentative capabilities of *R. albus* 7 on a wide range of fibrous substrates. Analysis of the *R. albus* 7 transcriptome suggests that it initiates a broad transcriptional response to growth on cellulose, including an increase in the tryptophan biosynthetic operon and a range of CBM37-containing coding regions. Our data suggest that *R. albus* 7 relies heavily on CBM37-containing proteins to coordinate a fibrolytic response instead of using cellulosomes. We also found little evidence to support a role for pil-like proteins or glycocalyx components in the transcriptional response of *R. albus* 7 grown on cellulose. Taken together, our findings reveal unique aspects of fiber degradation by *R. albus* 7 and contribute to a revised model for the cellulolytic strategy employed by this important ruminococcal species.

## Methods

### Growth conditions

The culture medium contained the following (per liter) [45]: 1.50 g KH_2_PO_4_, 1.13 g NaCl, 0.91 g NH_4_Cl, 5.0 g Na_2_CO_3_, 0.11 g MgCl_2_^.^6H_2_O, 0.082 g CaCl_2_^.^2H_2_O, 0.026 g FeSO_4_^.^7H_2_O, 0.032 g MnCl_2_^.^4H_2_O, 0.0024 g CoCl_2_^.^6H_2_O, 0.0022 g ZnCl_2_, 1.13 g yeast extract, 0.004 g 3-phenylpropionic acid, 0.002 g resazurin, 12.5 mL vitamin mix (mg per L: 20 thiamine HCl, 20 Ca-D-pantothenate, 20 nicotinamide, 20 riboflavin, 20 pyridoxine HCl, 1.0 p-aminobenzoic acid, 0.5 biotin, 0.2 vitamin B12, 0.125 folic acid, 0.124 tetrahydrofolic acid ), 8.4 mL volatile fatty acid mix (per L: 10 mL of each isobutyric, 2-methylbutyric, isovaleric, and *n*-valeric acids pH to 7), 0.63 g cysteine HCl, and an energy source (5.36 g cellobiose or 5.0 g Sigmacell 50 microcrystalline cellulose; Sigma-Aldrich, St. Louis, MO). The fully reduced medium was inoculated with 10 mL of a *R. albus* 7 (ATCC 21270) culture grown for 24 h on the same medium. Cultures were grown at a dilution rate of 0.033 h^-1^ under continuous gassing with CO_2_ at 39°C in a 760 mL bioreactor employing a segmented slurry delivery system [45]. After reaching steady state (>3 volumetric dilutions) samples were withdrawn once daily for 3 or 4 days for RNA extraction and chemical analysis.

### Chemical analysis

Samples (1500 μL) were centrifuged (12,000 × *g*, 5°C, 10 min) and culture supernatants were assayed for soluble sugars using the phenol-sulfuric acid method [46] with glucose as a standard. Fermentation products were determined by HPLC [45]. Cell pellets were washed with saline (9 g NaCl L^-1^), lyophilized, resuspended in 0.2 *N* NaOH and boiled for 30 min. The resulting cell lysates were clarified by centrifugation and protein content was determined [47]. For chemostat cultures grown with cellulose, an additional culture sample (~20 mL, weighed to 0.001 g) was collected each day and residual cellulose was determined gravimetrically as acid-detergent fiber [48] and the detergent-treated residue was collected on GF/D glass fiber filters (Pall Gelman, Port Washington, NY).

### RNA isolation

RNA was extracted from 9 mL of culture using a phenol-chloroform extraction followed by ethanol precipitation [49]. Ribosomes were removed from the RNA samples using a RiboZero Gram-positive rRNA removal kit (Epicentre, Madison, WI) according to manufacturer’s protocol. All samples were then barcoded, library prepared, and sequenced using a single channel of a flow cell on an Illumina HiSeq 2000 Sequencer at the University of Wisconsin-Madison Biotechnology Center.

### Polysaccharide hydrolysis and growth measurements

Experiments were conducted under a CO_2_ gas phase in triplicate 60 mL glass serum vials that contained 10 mL of modified Dehority medium [45] supplemented with the indicated polysaccharide in Table 1. Cultures were incubated without shaking at 39°C for 72 h. Hydrolysis of polysaccharides was measured as the release of reducing sugars by the dinitrosalicylic acid method [50], using glucose as a standard. Growth on polysaccharides was not measured directly, but was instead assumed from measurement of product (viz., ethanol and acetate) formation [51], using HPLC [45].

### Ortholog analysis and CAZyme annotation

Predicted protein sequences from one complete and seven draft ruminococcal genome sequences (Figure 1) were obtained from the National Center for Biotechnological Information (NCBI) and combined into one file. These include *R. albus* 7 (Accession: PRJNA51721, complete), *R. albus* 8 (PRJNA47357, draft), *R. flavefaciens* FD-1 (PRJNA55965, draft), *R. bromii* L2-63 (PRJNA197158, draft), *R. lactaris* ATCC 29176 (PRJNA54903, draft), *R. torques* ATCC 27756 (PRJNA54511, draft), *R. gnavus* ATCC 24149 (PRJNA54537, draft), and *R. obeum* ATCC 29174 (PRJNA54509, draft). Protein pairs and their similarity scores were identified using the OrthoMCL Algorithm [37] in a series of steps outlined as described in the OrthoMCL software version 2.0 guide. The protein pairs were clustered using the Markov Cluster Algorithm [52]. From each cluster, we chose one representative sequence per organism, based on the sequence that produced the highest aggregate blast bit score when blasted against every other sequence. The bit scores were used to eliminate paralogs. Using these representative sequences, we determined the number of sequences that were unique and those that were shared between all organisms. The representative sequences from each cluster were annotated for Carbohydrate-active Enzymes (CAZy) using the CAZy Analysis Toolkit (CAT) (http://mothra.ornl.gov/cgi-bin/cat.cgi; Pfam rule based annotations) [53].

### Cellulosome component analysis

CAZyme annotations for the predicted proteomes from ten Clostridiales genome sequences were obtained from the CAZy database (http://www.cazy.org/) [54]. The organisms analyzed include: *C. thermocellum* ATCC 27405*, C. cellulovorans* 743B*, C. cellulolyticum* ATCC 35319*, R. flavefaciens* FD-1, *C. phytofermentans* ISDg, *R. albus* 7, *R. albus* 8, *C. perfringens* ATCC13124*, R. torques* L2-14*,* and *R. bromii* L2-63. CAZyme annotations pertaining to known cellulases (GH5, GH8, GH9, GH12, GH44, GH48, GH74, and GH124) were selected [55]. Annotations corresponding to known dockerin (IPR01824, IPR016134, IPR002195) and cohesin (IPR002102 and IPR018452) domains were obtained from Interpro [56] through the DOE JGI IMG database [57]. A cellulosome-component matrix was constructed based on total counts for each annotation for all genomes, with each row corresponding to each of the ten organisms and each column a given cellulosomal annotation. A similarity matrix was constructed by calculating the correlation between all organismal pairs using Spearman's rank correlation (with tie-correction). A dendrogram was generated from the similarity matrix using the neighbor-joining program that is part of the PHYLIP package (http://evolution.genetics.washington.edu/phylip.html). A second identical analysis was performed where total counts for each annotation were normalized against the number of predicted proteins in each respective genome, producing an identical dendrogram.

### RNAseq analysis

A single FASTA file containing the *R. albus* 7 chromosome and its four plasmids was obtained from the NCBI (Accession: PRJNA51721). A FASTA files containing reads obtained from our RNA-seq experiments were aligned to the reference genome using the Burrows-Wheeler Aligner (BWA) [58], an alignment algorithm that allows for gaps (indels), using the default settings. The resulting SAM files were converted into sorted and indexed BAM files using SAMtools [58]. Additional file 1: Table S3 contains the total number of reads for each sample and reads aligned to the *R. albus* 7 genome by BWA.

We counted the number of reads per gene using tools from the open-access RsamTools package [59], an add-on library to the freely-available statistical software and programming language R [60]. Analysis of differential expression was done using EBSeq [61] and DESeq [62]. Median normalization technique of Deseq was used to account for differences in sequencing depth. Results generated using EBSeq are reported here as a ‘posterior probability of differential expression’ (PP of DE) for each of the 3,872 genes on the central chromosome and four plasmids. Genes were declared differentially expressed at a false discovery rate controlled at 100x (1-α) % by taking all genes with PP of DE greater than 1 - α.

#### RT-qPCR validation of RNAseq results

Total RNA from the chemostat samples subjected to RNA-seq (3 cellulose and 2 cellobiose) were reverse transcribed using iScript RT enzyme, according to the manufacturer’s protocol (Biorad, USA). The resulting cDNA was used to quantify the Rumal_1716 and 16S rRNA genes by amplification using the iQ Sybr Green Supermix according to the manufacturer’s protocol (Biorad, USA) on a BioRad CSX-Connect qPCR machine. Amplification of Rumal_1716 and 16S rRNA was performed using the following primer pairs: Rumal_1716*:* ATGCCGTTAAGGAAGCG and CACACCTATCGCCTGATA; and 16S rRNA: CCCTAAAAGCAGTCTTAGTTCG and CCTCCTTGCGGTTAGAACA. Relative expression (fold change) was calculated for Rumal_1716, normalized to the 16S rRNA gene, between cellulose and cellobiose samples. No amplification was observed in our negative controls, which consisted of samples not treated with reverse transcriptase and samples with nucleic acids omitted.

#### Quantitation of tryptophan

*In silico measurement of tryptophan enrichment of CAZymes*. Amino acid sequences for each gene in a given genome were downloaded in FASTA format from the NCBI database (http://www.ncbi.nlm.nih.gov/). The Reference Accessions for each predicted CAZyme in a given genome were obtained from the CAZY database (http://www.cazy.org). and submitted to the NCBI Batch Entrez database (http://www.ncbi.nlm.nih.gov/sites/batchentrez) to obtain the related amino acid sequences. Total amino acid counts, as well as counts for tryptophan, tyrosine, and methionine were tabulated within the entire genome and compared to the counts within the subset of CAZymes. Fisher’s exact test was used to calculate statistical significance for over- and under-enrichment.

*Quantification of extracellular tryptophan. R. albus* 7 was grown in triplicate in 100 mL of modified Dehority medium [45] on cellulose or cellobiose. Resazurin was omitted from the medium because it interfered with the colorimetric assay. After 30 h of growth (mid-exponential phase), cells were removed by centrifugation for 20 minutes at 4000 x *g* and spent media was concentrated 50-fold by lyophilization. Concentrated spent media (235 μL) was analyzed in a 0.5 mL reaction containing 3.67% formic acid and 6 N HCl in a glass vial. Absorbance at 595 nm was measured after incubation at 50°C for 48 hours and compared to a standard curve of pure L-tryptophan prepared in modified Dehority medium. The limit of detection by this assay was 0.1 mM.

#### Chemicals

Sigmacell 20 microcrystalline cellulose, cellobiose, larchwood xylan, lichenan, amylose (Type III), inulin (from chicory) and citrus pectin were from Sigma-Aldrich. Laminarin was from United States Biochemical. Curdlan was from Wako Pure Chemical Industries. Homoxylan was purified from tobacco stalks as described previously [63].

## Availability of supporting data

The data sets supporting the results of this article are included within the article and its additional supplementary file. All reads and the final transcriptome described in the manuscript are available in the GenBank repository under BioProject accession PRJNA238076 at http://www.ncbi.nlm.nih.gov/bioproject/238076.

## Electronic supplementary material

Additional file 1: **Supplementary Information 1.** Genes with lower transcription during growth of *Ruminococcus albus* 7 on cellulose compared to cellobiose. **Table S1.** Ortholog clusters with CAZy annotations among cellulolytic ruminococci. **Table S2.** Ethanol yields by model organisms in batch culture. **Table S3.** Total and aligned RNAseq reads. **Table S4.** Genes with 4-fold lower transcription during growth of *Ruminococcus albus* 7 on cellulose compared to cellobiose as the sole carbohydrate source assessed by RNA-Seq. **Figure S1.** Graphical representation of differential expression (DE). **Figure S2.** Expression of genes from each biological replicate. **Figure S3.** Putative pil/sec locus in *Ruminococcus albus* 7 is not transcriptionally changed by growth on cellulose compared to cellobiose. **Figure S4.** Putative biosynthetic genes for glycoside components of the glycocalyx were not significantly upregulated by growth on cellulose in *Ruminococcus albus* 7. (DOC 493 KB)
