# Supplementary material for: Unique aspects of fiber degradation by the ruminal ethanologen Ruminococcus albus 7 revealed by physiological and transcriptomic analysis
Source: BMC Genomics. 2014 Dec 4;15(1):1066. doi: 10.1186/1471-2164-15-1066 (PMC4300822; doi:10.1186/1471-2164-15-1066)
Supplement: Supplementary file 1 — Additional file 1: Supplementary Information 1. Genes with lower transcription during growth of Ruminococcus albus 7 on cellulose compared to cellobiose. Table S1. Ortholog clusters with CAZy annotations among cellulolytic ruminococci. Table S2. Ethanol yields by model organisms in batch culture. Table S3. Total and aligned RNAseq reads. Table S4. Genes with 4-fold lower transcription during growth of Ruminococcus albus 7 on cellulose compared to cellobiose as the sole carbohydrate source assessed by RNA-Seq. Figure S1. Graphical representation of differential expression (DE). Figure S2. Expression of genes from each biological replicate. Figure S3. Putative pil/sec locus in Ruminococcus albus 7 is not transcriptionally changed by growth on cellulose compared to cellobiose. Figure S4. Putative biosynthetic genes for glycoside components of the glycocalyx were not significantly upregulated by growth on cellulose in Ruminococcus albus 7. (DOC 493 KB) [file 12864_2014_6790_MOESM1_ESM.doc]

**Supplementary Information 1. Genes with lower transcription during growth of *R. albus* 7 on cellulose compared to cellobiose.**

Compared to the broad overexpression of genes in response to cellulose, only 2.5% of genes had lower transcription during growth on cellulose and only 16 genes with greater than 4-fold lower transcription (Supplementary Table 4). Transcripts encoding biotin synthesis proteins, along with the biotin transporter BioY, were decreased greater than 4-fold in *R. albus 7* cultures grown on cellulose as compared to cellobiose. Biotin is a cofactor involved in the transfer of C_1_ groups. However, transcripts encoding the synthetic enzymes of other cofactors involved in the transfer of C_1_ groups, including S-adenosylmethionine and tetrahydrofolate, were not differentially expressed on cellulose as compared to cellobiose (data not shown). As a component of our vitamin mix present in the growth media, 5.9 μg/L (or ~24 nM) of biotin was present under all conditions tested. Therefore, it is unlikely that this decrease in transcription of biotin synthetic genes was in response to changes in biotin concentration, but rather, may indicate a metabolic shift in response to growth on cellulose.

**Supplementary** **Table 1. Ortholog clusters with CAZY annotations among cellulolytic ruminococci.**

| **Organism(s)** | **Cazy family^1^** | **Representative loci** |
| --- | --- | --- |
| *R. albus* 8 | (no unique clusters with Cazy annotations) | |
| *R. albus* 7 | GH27 | YP_004104702 |
| *R. flavefaciens* FD-1  (91 total) | GH16 | ZP_06142932 |
|  | GH3 | ZP_06144849 |
|  | GH97 | ZP_06144890 |
|  | GT51 | ZP_06142823 |
|  | CE1 | ZP_06144944 |
| *R. albus* 7 and  *R.* *albus* 8  (631 total) | CBM13 | ZP_08160059 |
|  | CBM13/GH43 | ZP_08160455 |
|  | CBM4/CBM9/CBM16/GH10/CBM22 | ZP_08159615 |
|  | CBM4/CBM9/GH43/CBM16/CBM22 | ZP_08160002 |
|  | CBM50 | ZP_08158039 |
|  | CBM50 | YP_004105654 |
|  | CE6 | ZP_08159537 |
|  | CE8 | ZP_08159475 |
|  | GH10 | YP_004104735 |
|  | GH130 | ZP_08159838 |
|  | GH19 | ZP_08159012 |
|  | GH25 | ZP_08157437 |
|  | GH25 | ZP_08157332 |
|  | GH26 | ZP_08157423 |
|  | GH26 | ZP_08159989 |
|  | GH3 | ZP_08160226 |
|  | GH48 | ZP_08158497 |
|  | GH5 | ZP_08160422 |
|  | GH5 | ZP_08158504 |
|  | GH5 | ZP_08159132 |
|  | GH5 | ZP_08157835 |
|  | GH5 | ZP_08157350 |
|  | GH5 | ZP_08160335 |
|  | GH5 | ZP_08160389 |
|  | GH5 | ZP_08158005 |
|  | GH5 | ZP_08159293 |
|  | GH67 | ZP_08159559 |
|  | GH9 | ZP_08159634 |
|  | GT2 | ZP_08160459 |
|  | GT2 | ZP_08160477 |
|  | GT2 | ZP_08159287 |
|  | GT4 | ZP_08160438 |
|  | GT4 | ZP_08160444 |
| *R. albus* 7 and  *R. flavefaciens* FD-1  (130 total) | CE1 GH11 | ZP_06142343 |
|  | GT2 | ZP_06142272 |
|  | GH5 | ZP_06143262 |
|  | PL1 | ZP_06142855 |
|  | GH9 | ZP_06142678 |
|  | GH43 | ZP_06143103 |
|  | GH5 | ZP_06141809 |
| *R. albus* 8 and  *R. flavefaciens* FD-1  (90 total) | GH43 | ZP_08160877 |
|  | CE1 | ZP_08157508 |
|  | CE10 | ZP_08157752 |
| *R. albus* 7,  *R.* *albus* 8, and  *R. flavefaciens* FD-1  (297 total) | CBM13/GH43 | ZP_06141808 |
|  | CBM32 | ZP_06143078 |
|  | CBM4/CBM9/CBM16/GH10/CBM22 | YP_004104519 |
|  | CBM4/CBM9/CBM16/GH11/CE4/CBM22 | YP_004104068 |
|  | CE9 | ZP_06144710 |
|  | CE9 | ZP_08157504 |
|  | GH11 | ZP_06142259 |
|  | GH127 | ZP_06142964 |
|  | GH130 | ZP_08158270 |
|  | GH16 | ZP_08159157 |
|  | GH25 | YP_004105733 |
|  | GH26 | YP_004106251 |
|  | GH26 | ZP_06144005 |
|  | GH26 | ZP_08158982 |
|  | GH3 | ZP_06143884 |
|  | GH3 | YP_004106017 |
|  | GH44 | ZP_08159205 |
|  | GH5 | YP_004103747 |
|  | GH53 | ZP_08157980 |
|  | GH9 | ZP_06142598 |
|  | GH9 | ZP_06142593 |
|  | GH9 | YP_004104210 |
|  | GT2 | ZP_06142487 |
|  | GT2 | YP_004103491 |
|  | GT4 | ZP_06143341 |
|  | PL10/CE8 | ZP_08159991 |

^1^CAZymes with predicted cellulase activity are shaded.

**Suppementary Table 2. Ethanol yields by model organisms in batch culture.**

| **Organism** | **Ethanol (g/g)** | **Substrate** | **Citation** |
| --- | --- | --- | --- |
| *Clostridium thermocellum* ATCC27405 | 0.184 | crystalline cellulose |  |
| *Clostridium phytofermentans* ISDg | 0.175 | treated plant material |  |
| *Trametes hirsuta* WT | 0.17 | cellulose |  |
| *Saccharomyces cerevisiae* EBY100^recomb^ | 0.31 | hemicellulose |  |
| *Escherichia coli* KO11^recomb^ | 0.42 | glucose |  |
| *Escherichia coli* BL21 pLysS^recomb^ | 0.144 | hemicellulose |  |
| *Escherichia coli* ATCC8739^recomb^ | 0.281 | brown microalgae |  |
| *Ruminococcus albus 7* | 0.179 | crystalline cellulose | This study |

**Supplementary Table 3. Total and aligned RNAseq reads.**

| Sample | **Cellulose 1** | **Cellulose 2** | **Cellulose 3** | **Cellobiose 1** | **Cellobiose 2** | **Cellobiose 3** |
| --- | --- | --- | --- | --- | --- | --- |
| Total Reads | 15,910,389 | 18,290,170 | 14,886,674 | 9,945,782 | 9,801,027 | 10,499,563 |
| Aligned Reads | 14,791,315 | 16,972,608 | 13,857,781 | 9,171,099 | 6,929,484 | 9,717,517 |
| Percent Aligned | 93.0 | 92.8 | 93.1 | 92.2 | 70.7 | 92.6 |

**Supplementary Table 4. Genes with 4-fold lower transcription during growth of *Ruminococcus albus 7* on cellulose compared to cellobiose as the sole carbohydrate source assessed by RNA-Seq.**

| Gene (Rumal_) | Annotation | Fold Change^α^ | Signal sequence^β^ |
| --- | --- | --- | --- |
| 0999 | hypothetical protein | 0.04 | No |
| 0998 | glutamate dehydrogenase (NADP(+)) | 0.05 | No |
| 1684 | adenosylmethionine-8-amino-7-oxononanoate aminotransferase | 0.11 | No |
| 1685 | dethiobiotin synthase | 0.13 | No |
| 1686 | biotin synthase | 0.14 | No |
| 1687 | BioY protein | 0.14 | Sec |
| 3691 | BioY protein | 0.17 | TM |
| 3363 | hypothetical protein | 0.19 | No |
| 2194 | hypothetical protein | 0.22 | Sec |
| 2175 | glucuronate isomerase | 0.23 | No |
| 0713 | hypothetical protein | 0.23 | No |
| 2193 | hypothetical protein | 0.24 | No |
| 2177 | mannitol dehydrogenase domain | 0.24 | No |
| 2191 | hypothetical protein | 0.25 | TM |
| 2601 | Pectate lyase/Amb allergen | 0.25 | Sec |
| 2178 | ABC transporter-like protein | 0.26 | No |

^α^ Each gene reported here was significantly DE, with a PP of DE greater than 0.95

^β^ Predicted signal sequence predicted by PRED-TAT TM=transmembrane.

**Supplementary Figure 1. Graphical representation of differential expression (DE).** PP of DE shown as fold changes (Fcu) in gene expression during growth of *R. albus* 7 on cellulose compared to cellobiose on the log_2_ scale across the length of the entire chromosome (x-axis). Bars are shaded gray (not DE at 1% false discovery rate), orange (DE at 1% false discovery rate) or red (DE at 0.01% false discovery rate).

**Supplementary Figure 2. Expression of genes from each biological replicate**. Total number of transcripts from three independent biological replicates grown on cellulose (left column) or cellobiose (right column) were mapped onto the genome of *R. albus* 7. Dotted lines indicate the start/end of the chromosome and 4 plasmids. The Cellob-2 sample was not included in further analysis due to a suspected nucleotidase contamination during sample preparation.

**Supplementary Figure 3. Putative *pil/sec* locus in *R. albus* 7 is not transcriptionally changed by growth on cellulose compared to cellobiose.** This 3.9 kb region contains two putative *pil* genes, Rumal_0365 and Rumal_0366, and two putative protein-export membrane proteins, Rumal_0367 and Rumal_0368. Genes are represented by boxes, putative annotations are shown in parentheses. Average transcriptional fold-change of these genes on cellulose compared to cellobiose is shown in bold.

**Supplementary Figure 4. Putative biosynthetic genes for glycoside components of the glycocalyx were not significantly upregulated by growth on cellulose in *R. albus* 7**. Predicted pathways for sugar components of the glycocalyx (glucose, xylose, mannose, and fructose) are shown. Intermediates are in boxes, enzyme names are italicized. Where found, *R. albus* 7 genes with relevant annotated functions are indicated by Rumal_ number. Average fold-change in transcription during growth on cellulose (compared to growth on cellobiose) for each *R. albus* 7 gene is indicated in parentheses. Abbreviations: P=phosphate; UDP= uridine diphosphate; GDP= guanosine diphosphate.
